# Assessment of the length of sick leave in patients with ischemic heart disease

**DOI:** 10.1186/s12872-016-0460-7

**Published:** 2017-01-18

**Authors:** Nausica Català Tella, Catalina Serna Arnaiz, Jordi Real Gatius, Oriol Yuguero Torres, Leonardo Galván Santiago

**Affiliations:** 1 ABS Eixample, Institut Català de la Salut, Balmes avenue, 4, 25006 Lleida, Spain; 2 Institut Universitari d’Investigació en Atenció Primària Jordi Gol (IDIAP Jordi Gol), Lleida, Spain; 3 Facultat de Medicina i Ciències de la Salut, Universitat Internacional de Catalunya, Sant Cugat, Barcelona, Spain; 4 Servicio de Urgencias del Hospital Universitari Arnau de Vilanova, Lleida, Spain; 5 Departament Català de la Salut, Servicio de farmacia, Lleida, Spain

**Keywords:** Sick leave, Ischemic heart disease, Acute coronary syndrome, Coronary heart disease, Acute myocardial ischemia, Cost, Anxiety/depression

## Abstract

**Background:**

The prevalence of ischemic heart disease is high. Few recent studies have investigated the periods of sick leave of these patients. Our aim is to determine the length of sick leave after an acute coronary syndrome, its costs, associated factors and to assess the use of antidepressants and/or anxiolytics.

**Methods:**

An observational study of a retrospective cohort of patients on sick leave due to ischemic heart disease in a health region between 2008–2011, with follow-up until the first return to work, death, or end of the study (31/12/2012). Measurements: length of sick leave, sociodemographic variables and medical prescriptions.

**Results:**

Four hundred and ninety-seven patients (mean age 53 years, 90.7% male), diagnosed with acute myocardial infarction (60%), angina pectoris (20.7%) or chronic form of ischemic heart disease (19.1%). Thirty-seven per cent of patients took anxiolytics the year after diagnosis and 15% took antidepressants. The average duration of sick leave was 177 days (95% CI: 163–191 days). Patients diagnosed with acute myocardial infarction returned to work after a mean of 192 days, compared to 128 days in cases with angina pectoris. Patients who took antidepressants during the year after diagnosis returned to work after a mean of 240 days. The mean work productivity loss was estimated to be 9,673 euros/person.

**Conclusions:**

The mean duration of sick leave due to ischemic heart disease was almost six months. Consumption of psychotropic medication doubled after the event. Older age, suffering an acute myocardial infarction and taking antidepressants were associated with a longer sick leave period.

## Background

In recent decades, mortality rates after Ischemic Heart Disease (IHD) have declined in developed countries, but IHD nonetheless remains the leading cause of death in men and the second most frequent in women [1]. A study in the primary care setting places the prevalence of IHD in the general population over 15 years at 5.5% [1]. The mortality rate of patients with Acute Coronary Syndrome (ACS) in the first month is 50%; almost half of these deaths occur in the first two hours, mainly due to ventricular fibrillation [2]. This high prehospital mortality has not changed in recent years, although hospital mortality has decreased notably [2].

It is estimated that every year cardiovascular disease causes over 4 million deaths in Europe as a whole, and 1.9 million deaths in the European Union, most due to Coronary Heart Disease [3]. From an epidemiological point of view, IHD is particularly important in our environment due to its high prevalence, the high consumption of resources it requires, and the associated mortality. The increase in its prevalence and advances in technology have established IHD as the disease with the greatest economic impact in the developed countries [4]. Incapacity for work is one of the indirect costs of the disease, but this issue has not been widely studied [4–6]. Few authors have assessed the length of periods of sick leave due to IHD [2, 7–10] since the review of the topic conducted in 2004 by Perk and Alexanderson [11]. The study by Sicras-Mainar in 2009 quantified the cost per patient following an episode of ACS at 14,069 euros (87% of which were direct costs and 13% lost productivity costs). The direct costs comprised primary care (20%) and specialized care (67%), with hospitalization costs accounting for 63% of the total [5].

Advances in the management of the acute phase of ACS and of heart failure and revascularization in angina pectoris, among other conditions, have improved prognosis and recovery time. These developments may potentially allow a much earlier return to work.

Incapacity for work is one of the major contributing factors to the economic burden of a disease. If current healthcare systems are to remain viable, clinicians, economists, health authorities and the biomedical industry must work together to establish a consensus on issues such assustainable periods of sick leave indifferent clinical situations [4].

The present study was designed to determine the mean length of sick leave after an ACS. We also aimed to evaluate some of the associated factors such as age, sex, specific diagnosis andthe prescription of medicationfor depression or anxiety, and to estimate the indirect costs of incapacity for work associated with ischemic heart disease.

## Methods

This observational, retrospective, longitudinal study examined data from the primary care clinical records of patients in the health region of Lleida between 1 January 2008 and 31 December 2011. Data were extracted on all patients aged between 18 and 65 years diagnosed with IHD and on sick leave taken forthis condition. Patients’ evolution was followed until December 31, 2012.

### Data source

The data were obtained from the medical histories in the database of the Catalan Health Service primary care section. In Catalonia, the use of the health card is mandatory to obtain medication through the Social Security system. The sedatives prescribed corresponded to the groups N03AE/N05BA-BB-BX-CD-CF-CM and the antidepressants to the groups N06A/N06B/N06C in the Anatomical Therapeutic Chemical classification used in Spain [12].

Date of birth, sex, employment, exitus (if appropriate) and diagnosis of IHD were extracted from the primary care information systems in accordance with the International Classification of Diseases-10 (ischemic heart disease: I20-I25).

### Variables

The variables assessed were length of sick leave period (calculated from the beginning of the leave until the return to work, or until 31/12/2012 or date of death), age at the beginning of sick leave, sex, clinical diagnosis of IHD, and the prescription of antidepressants and/or anxiolytics.

### Patient selection criteria

Patients with incapacity due to IHD recorded between 1 January 2008 and 31 December 2011, aged 18 to 65 at the time of diagnosis, and living in the Lleida health region were included.

### Non-healthcare costs

Indirect costs were those relating to productivity loss (number of periods of sick leave and total days off work). They were quantified according to the minimum wage (Source: Spanish Institute of Statistics), considering the cost per day not worked at 54.65 euros [5]. Indirect costs were estimated for the whole of the patients’ follow-up period.

### Statistical analysis

Descriptive analysis of the general characteristics of the cohort, expressing qualitative variables as frequencies and percentages and quantitative variables as means and standard deviations (SD). The duration of sick leave was estimated by survival analysis until the return to workusing the Kaplan-Meier method. The length of periods of sick leave was compared using the Log-Rank hypothesis test. At multivariate level, Hazards ratios for return to workwere estimated from the fit of the multivariate Cox regression models. The assumption of proportional hazards of the Cox models was evaluated using Schofield residuals. Data were analysed using the IBM SPSS statistical package (version 19). The main results are expressed with 95% confidence intervals (95%) and *p*-values less than 0.05 were considered statistically significant.

## Results

Of a total of 497 patients with incapacity due to IHD, 90.7% were male. The most common diagnosis was acute myocardial infarction (AMI - 59.4%), followed by angina pectoris (20.7%) and chronic forms of IHD (19.1%). The remaining 0.8% were due to other acute ischemic heart diseases and complications after AMI. The average age was 53 years (SD: 7.4). Broken down by years, 142 individuals were diagnosed with IHD (28.6%) in 2008; 117 (23.5%) in 2009; 112 (22.5%) in 2010, and finally 126 (25.4%) in 2011. Anxiolytic treatment was required by 37% of patients within 12 months of diagnosis, and antidepressant treatment by 15% (Table 1).

**Table 1:** Descriptive data of the variables analysed

Variables	Number	Percent
Diagnostic code (CIE10) and description		
Angina (I20)	103	20.70%
Acute myocardial infarct (AMI) (I21)	295	59.40%
Complications following AMI (I23)	1	0.20%
Other acute ischemic heart disease (I24)	3	0.60%
Chronic ischemic heart disease (I25)	95	19.10%
Age group		
<=45 years	88	17.70%
46 to 55	195	39.20%
> = 56	214	43.10%
Year of sick leave		
2008	142	28.60%
2009	117	23.50%
2010	112	22.50%
2011	126	25.40%
Sex		
Female	48	9.70%
Male	449	90.30%
Medication dispensed : Yes/No ATC code)		
Prior sedative (N03AE N05BA-BB-BX-CD-CF-CM)		
Previous	136	27.4%
Previous 6 months	73	14.7%
Previous 12 months	95	19.1%
Subsequent 12 months	184	37.0%
Antidepressants GT = N06A / N06B / N06C		
Previous	74	14.9%
Previous 6 months	30	6.0%
Previous 12 months	39	7.8%
Subsequent 12 months	76	15.3%

The estimated mean length of sick leave was 177 days (95% CI 163–191). In women it was 152 days compared with 180 days in men, though the difference was not statistically significant (*p* = 0.22). Half of the participants were on sick leave for less than four months.

**Fig. 1:**
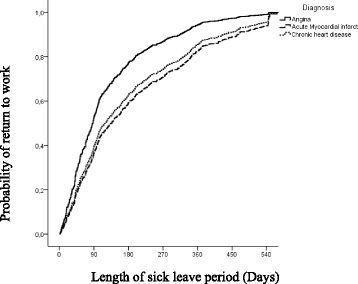
Probability of return to work in relation to the length of sick leave period

The length of the sick leave period increased with age, as the older group (≥56 years) had a mean sick leave of 186 days (≈6 months) compared with 140 days (≈4.5 months) in the ≤45 year group (*p* <0.05) (Table 2). Patients with angina pectoris were the first to return to work (Fig. 1); on average, patients diagnosed with AMI and IHD returned to work 60 days (*p* = 0.002). Finally, patients who required antidepressant medication in the 12 months after diagnosis had longer periods of sick leave (*p* <0.05) than those who did not (Table 2).

**Table 2:** Estimated time in days of the overall period of sick leave according to group

			95% CI			95% CI		*p*-value
Variable		Mean	(Upper limit -	Lower limit)	Median	(Upper limit -	Lower limit)	
Global		177.3	(163.5-	191.0)	116	(98.6-	133.4)	
Diagnostic code (CIE10) and description								0.002
Angina (I20)		127.8	(99.9-	155.6)	68	(46.6-	89.4)	
Acute Myocardial Infarction (I21)		191.8	(174.1-	209.5)	131	(102.1-	159.9)	
Other acute ischemic heart disease (I24)		143.0	(47.8-	238.2)	187	(0.0-	412.6)	
Chronic ischemic heart disease (I25)		187.7	(154.5-	220.9)	137	(105.7-	168.3)	
Age group (in years)								0.042
<=45 years		139.7	(110.2-	169.2)	88	(56.7-	119.3)	
46 to 55		184.4	(161.7-	207.1)	125	(98.4-	151.6)	
> = 56		186.1	(165.2-	207.1)	137	(103.4-	170.6)	
Sex								0.221
Female		152.5	(110.7-	194.3)	99	(62.8-	135.2)	
Male		179.9	(165.3-	194.5)	119	(101.0-	137.0)	
Previous medication								
Previous sedative (N03AE N05BA-BB-BX-CD-CF-CM)	SI	189.6	(162.7-	216.6)	126	(80.3-	171.7)	0.35
	No	172.4	(156.5-	188.4)	112	(93.5-	130.5)	
Sedative in previous 12 months (N03AE N05BA-BB-BX-CD-CF-CM)	Si	208.4	(173.8-	243.0)	160	(84.5-	235.5)	0.045
	No	169.7	(154.9-	184.5)	111	(95.1-	126.9)	
Previous antidepressants: GT = N06A/N06B/N06C	Si	181.3	(146.5-	216.1)	137	(90.2-	183.8)	0.577
	No	176.5	(161.5-	191.4)	111	(93.6-	128.4)	
Antidepressants in previous 12 months : GT = N06A/N06B/N06C	Si	228.9	(174.0-	283.8)	191	(105.3-	276.7)	0.022
	No	172.8	(158.7-	186.9)	110	(92.7-	127.3)	
Subsequent medication (12 months)								
Sedatives	Si	199.3	(175.6-	223.0)	150	(113.5-	186.5)	0.015
	No	164.2	(147.5-	180.9)	103	(88.9-	117.1)	
Antidepressants	Si	240.2	(199.3-	281.1)	196	(133.0-	259.0)	<0.001
	No	165.9	(151.6-	180.2)	108	(93.1-	122.9)

Table 3 shows the multivariate analysis of the hazards ratios for returning to work depending on the variables analysed. Delayed return to work was associated with the diagnosis of myocardial infarction, older age, and taking antidepressant treatment.

**Table 3:** Estimation of Hazard Ratio (HR) of return to work in relation to the variables analysed using the Cox regression model

Variable		95%CI		
Category	HR	(Lower level -	Upper level)	*p*-value
Sex				
Female	1.36	(1.00-	1.86)	0,051
Age in years	0.99	(0.97-	1.00)	0.025
Diagnosis (Ref: Chronic ischemic heart disease)				<0.001
Angina (CIE10:I20)	1.49	(1.12-	1.98)	0.006
Acute myocardial infarct (CIE10:I21)	0.91	(0.71-	1.15)	0.421
Medication previous 12 month (Ref: None)				
Sedative (N03AE N05BA-BB-BX-CD-CF-CM)	0.88	(1.08-	0.72)	0.217
Antidepressants GT = N06A / N06B / N06C	0.64	(0.84-	0.48)	0.002

In 12 of the 497 cases studied, the full sick leave period was not recorded. In five cases this was because of the finalization of the study on 31/12/2012; one patient moved to another centre, and six died.

The mean indirect cost due to loss of work productivity was estimated at 9,673 euros.

## Discussion

Patients who take sick leave due to IHD are predominantly male, and more than half havediagnoses of AMI. As regards medication, more than a third of these patients take anxiolytic treatment a year after diagnosis, and 15% antidepressants. In fact, patients’ consumption of anxiolytics or antidepressants doubles after the cardiovascular event. The mean period of sick leave is 177 days; the figure increases with age and in patients diagnosed with AMI. Patients receiving antidepressants have the longest mean period of sick leave (233 days).

Most patients on sick leave are male. Recent survey-based studies in the US continue to show a predominance of males in heart disease in general and in AMI in particular [13, 14]. Among the 40–59 year age group, the prevalence is 3.3% in males and 1.8% in females [13]. No recent data are available on the prevalence of heart disease in our country, but an indirect estimation can be made using data from the population surveys carried out by the National Institute of Statistics [13, 15]. In the last two surveys, which referred explicitly to AMI, estimates for each age group were well below those recorded in the US population, although males predominated in this country as well (0.64% in men vs. 0.15% in women in the 45–54 year age group 1.84% in men and 1.28% in women in the 55–64 year age group). In fact, ever since the Framingham cohort’s study it has been acknowledged that the incidence of coronary events increases rapidly with age and those rates are lower in women than in men [13, 16, 17].

Although little recent information on the length of sick leave due to IHD is available in the literature, our results are consistent with the few reports published to date. Andrée et al. [18] reported that 2.2% of applications for sick leave conceded in Spain in 2009 were due to cardiovascular disease, and that the sick leave period lasted more than six months in 69.9% of these patients. As that study did not differentiate according to type of cardiovascular event, the results cannot be compared directly with ours, since we focused specifically on ischemic heart disease. Lopez de la Iglesia reported that, in most cases of IHD, return to work is possible within 90 to 180 days [2]. Other sources like the Spanish National Social Security Institute (INSS) set the mean time for return to work after AMI at 90 days, ranging between 60 and 120 days depending on the anatomical location of the event and the diagnosis, with 30 days for both angina pectoris and old myocardial infarction [10]. Other studies of sick leave in the general population report longer periods and a wide variability, ranging from 189 days in the study by Jiménez [8] to 244 days in the study by Gutiérrez Morlote [9] and to a mean of 255 days in the Andalusia study protocol [7].

After an extensive review of the bibliography, Perk & Alexanderson (2004) state that few articles of scientific quality specifically discuss the duration of sick leave due to coronary heart disease. Perk & Alexanderson say that in Sweden a sick leave of at least three months is common after AMI [11, 19], and that in several European countries and the US the median duration of sick leave in this situation is 60 days [20]. The review concludes that there is no evidence to support the differences in the sick leave period from an international perspective [11]. Factors related to the almost infinite peculiarities of job positions, considerations such as age, motivation, satisfaction with previous position, salary, trade, unemployment rates, comorbidity, or even economic aspects - which in some studies have proved more influential on return to work than clinical endpoints - are involved in the decision [21–23]. Lastly, an important aspect to be considered is the timing of the return to work. Levine et al. say that traditionally, there was a wide timeframe for return to work after an acute coronary event [9, 21, 24]; however, this recommendation is now obsolete as it does not take into account the major improvements in acute therapies, preventive treatments and cardiac rehabilitation that have been achieved in recent years. Modern guidelines, though not entirely clear, have shortened this timeframe to 1–3 months [25].

The factor associated most clearly with longer sick leave was the concomitant use of antidepressant treatment. Previous studies have demonstrated the association between ischemic heart disease and depression: patients with cardiovascular disease are twice as likely to develop depression [26], and depression increases cardiovascular mortality and mortality due to any other cause [27], besides being associated with poorer prognosis and quality of life [26]. Consequently, several studies recommend that these patients should be screened for depressive symptoms [26, 28, 29] and stress the possible impact of depression on adherence to treatment [28]. It would also be interesting to determine whether early intervention to treat depression alters the evolution of the illness [26, 29] and whether this strategy shortens periods of sick leave in these patients.

Acute coronary syndrome has a high economic burden. The indirect costs associated with lost productivity, which according to a previous study represents only 13% of the total cost per person [5], are estimated at 9,673.05 euros per period of sick leave. For reference, according to the National Institute of Statistics, the Spanish minimum wage during the study period was 8,400 euros in 2008 and 8,979.6 euros in 2011 [30]. The 9,673 euros represent a 115 and a 108% of the minimum interprofesional salary in spain in year 2008 and 2011 respectively. Obviously, the direct costs must also be added to the equation.

The limitations of our study are mainly to do with the data collection, due to the possible loss of some prescriptions of antidepressant or anxiolytic medication supplied without a Social Security prescription orthrough other subsidized health insurance systems in Spain such as ISFAS and MUFACE. However we believe that the patients not covered by Social Security represent only a small proportion of the total and do not create a selection bias. We should also bear in mind that this is a descriptive study of the duration of sick leave for IHD which did not have access to other data that might influence the condition – for instance, cardiovascular or socioeconomic risk factors, or other prognostic risk factors associated with the disease such as inclusion in a rehabilitation program. On the other hand we are unable to include information on the severity of the event, which would have been very interesting. The patient’s type of employment should also be considered, in terms of the physical exertion required and the degree of associated stress. We would also like to have references from other diseases. Future studies should compare the economic impact of sick leave due to different pathologies using the same methodology (i.e., the same follow-up, the same context, and so on). These and other factors, in addition to the calculation of the total cost per patient (direct and indirect), should be included in future studies.

## Conclusions

Patients on sick leave due to ischemic heart disease spend an average of almost six months off work, a considerably longer period than that recommended by the Spanish Social Security system. This means that the indirect health costs of acute coronary syndrome are particularly high. Patients are mostly men, and the most frequent diagnosis is AMI. After the event, patients’ consumption of medication doubles, with over a third receiving anxiolytics and 15% antidepressants. The factors associated with a longer duration of incapacity are older age, acute myocardial infarction and receiving antidepressant treatment.

## Abbreviations

ACS: Acute coronary syndrome
AMI: Acute myocardial infarction
IHD: Ischemic heart disease
INSS: Spanish national social security institute
SD: Standard deviations
